# Microbial Ligand Costimulation Drives Neutrophilic Steroid-Refractory Asthma

**DOI:** 10.1371/journal.pone.0134219

**Published:** 2015-08-11

**Authors:** Sabelo Hadebe, Frank Kirstein, Kaat Fierens, Kong Chen, Rebecca A. Drummond, Simon Vautier, Sara Sajaniemi, Graeme Murray, David L. Williams, Pierre Redelinghuys, Todd A. Reinhart, Beth A. Fallert Junecko, Jay K. Kolls, Bart N. Lambrecht, Frank Brombacher, Gordon D. Brown

**Affiliations:** 1 Aberdeen Fungal Group, Infection, Immunity and Inflammation Programme, Institute of Infectious Disease and Molecular Medicine, Faculty of Health Science, University of Cape Town, South Africa; 2 International Centre for Genetic Engineering and Biotechnology and Division of Immunology, Institute of Infectious Disease and Molecular Medicine, Faculty of Health Science, University of Cape Town, South Africa; 3 VIB Inflammation Research Center, Laboratory of Immunoregulation and Mucosal Immunology, University Ghent, Ghent, Belgium; 4 Department of Paediatrics, University of Pittsburgh School of Medicine, Pittsburgh, Pennsylvania, United States of America; 5 Pathology, Division of Applied Medicine, Institute of Medical Sciences, Foresterhill, University of Aberdeen, Aberdeen, United Kingdom; 6 Department of Surgery and Center for Inflammation, Infectious Disease and Immunity, James H. Quillen College of Medicine, East Tennessee State University, Johnson City, Tennessee, United States of America; 7 Department of Infectious Diseases and Microbiology, Graduate School of Public Health, University of Pittsburgh, Pittsburgh, Pennsylvania, United States of America; 8 Department of Pulmonary Medicine, ErasmusMC, Rotterdam, The Netherlands; French National Centre for Scientific Research, FRANCE

## Abstract

Asthma is a heterogeneous disease whose etiology is poorly understood but is likely to involve innate responses to inhaled microbial components that are found in allergens. The influence of these components on pulmonary inflammation has been largely studied in the context of individual agonists, despite knowledge that they can have synergistic effects when used in combination. Here we have explored the effects of LPS and β-glucan, two commonly-encountered microbial agonists, on the pathogenesis of allergic and non-allergic respiratory responses to house dust mite allergen. Notably, sensitization with these microbial components in combination acted synergistically to promote robust neutrophilic inflammation, which involved both Dectin-1 and TLR-4. This pulmonary neutrophilic inflammation was corticosteroid-refractory, resembling that found in patients with severe asthma. Thus our results provide key new insights into how microbial components influence the development of respiratory pathology.

## Introduction

Asthma is a chronic respiratory disease characterised by variable and reversible airway obstruction, resulting from inflammation, mucus overproduction, and airway hyperresponsiveness (AHR) to inhaled stimuli. This causes recurrent episodes of wheezing, breathlessness, coughing and chest tightness [1]. The incidence of this disease, which is thought to affect over 300 million people worldwide, has increased dramatically, particularly in developed countries, but the reasons for this are unclear [2]. Although genetic components are certainly risk factors, the rapid rise in incidence precludes genetic changes in the population, and it is thought that alterations in exposure to environmental factors must be playing a major role. Indeed the “hygiene hypothesis” arose from the observation that the incidence of asthma was inversely correlated with exposure to infection [3].

Asthma is widely regarded as an allergic disease, involving the development of T helper 2 (Th2) adaptive responses and eosinophilic airway inflammation, and atopy is a strong predisposing factor for the development of asthma in children [4]. Clinically, however, the correlation between atopy and eosinophilic inflammation is less clear and it is now accepted that asthma is a heterogeneous disease involving several other non-allergic inflammatory conditions [5, 6]. Although the pathophysiological mechanisms underlying the various disease endotypes are unclear, there is growing evidence for an involvement of innate responses to microbes and their components in the pathogenesis of asthma [4]. Indeed, several components have already been implicated, including agonists of the Toll-like (TLR) and C-type lectin (CLR) receptors, such as lipopolysaccharide (LPS) and the fungal cell-wall component, β-glucan [7–10], respectively.

LPS and β-glucan have both been linked to the development of asthma in humans and shown to influence disease development in animal models [7, 11–14]. Importantly, costimulation with these agonists has been shown to have synergistic effects on inflammatory responses, particularly those favouring the development of Th17 responses [15–17]. Yet the contribution of such costimulatory responses on the development of pulmonary inflammation has not been explored, and was the focus of this study. We discovered that the combination of these agonists profoundly modulated allergic and non-allergic responses, promoting a Th-17-type neutrophilic inflammation that was corticosteroid resistant.

## Materials and Methods

### Mice

7–10 week old wild type (wt) and gene deficient mice (Dectin-1^-/-^, TLR-4^-/-^) in C57BL/6 or Balb/c background were bred and maintained in the specific pathogen-free facilities of the University of Aberdeen, University of Cape Town and University of Ghent. 1-Derβ TCR transgenic mice have been described previously [18]. All animal use was approved and in compliance with local University animal research ethical regulations and a UK Home Office project licence (60/4007). All experimental mice were sacrificed by CO_2_ asphyxiation at defined time points, as detailed in the text. The physical condition of the animals was checked daily.

### Airway challenges

In all experiments, mice were anaesthetised with a mixture of ketaset and xylazine (1:1) and compounds were administered intratracheally (i.t.) in 50 μl. After administration mice were roused with Antisedan.

For non-allergic challenges, mice were challenged i.t. on days 0, 3, 7 and 10 with LPS (100 ng; *Salmonella enterica*, L6143, Sigma-Aldrich, St. Louis, MO, USA, used in all experiments) and highly purified particulate β-glucan [19] (1x10^7^ particles, used in all experiments) alone or in combination, with PBS serving as control.

For allergic model involving parenteral sensitization with ovalbumin, mice were sensitized intraperitoneally (i.p.) with 200 μl of 25 μg LPS-free OVA (Hyglos GmbH, Munich, Germany) emulsified in 1 mg Alum (Invivogen, San Diego, CA, USA) on days 0 and 7 and then challenged i.t. on days 20, 21 and 22 with 50 μl of OVA alone (10 μg, used in all experiments) or with a combination of LPS and β-glucan. PBS served as control.

For experiments with house dust mite (HDM), mice were sensitized i.t. at day 0 and 7 with HDM alone (10 μg; Greer Laboratories, Lenoir, NC, *Dermatophagoides pteronyssinus* (Der p1) 145.56 mcg per vial, endotoxin 31.25 EU per vial, 2.87 mg protein per vial and 11.6 mg dry weight per vial) or with a combination of LPS and β-glucan. PBS was used a control. Mice were subsequently challenged i.t. on days 20, 21 and 22 with HDM (10 μg) or PBS. Lungs, bronchoalveolar lavage fluid (BALF) and mediastinal lymph nodes (MLN) were harvested 24 hrs post last challenge. In some experiments, mice were additionally treated with dexamethasone 21-phosphate disodium salt (Sigma-Aldrich, St. Louis, MO, USA) i.p. (3 mg/kg in 100 μl) on days 20, 21 and 22.

In all experiments BALF was isolated from sacrificed mice 24 hrs post last-challenge. Cells in isolated BALF were counted and identified by flow cytometry, as described below. Cytokine measurements were performed using Bio-Plex Pro Mouse cytokine 23-plex Assay (Bio-Rad Laboratories Ltd, USA), according to the manufacturer’s specifications.

Total IgE and HDM-specific IgE was measured in the serum as described previously [20, 21]

### Histology

Lungs were inflated with OCT (Tissue-Tek, Sakura, Netherlands) and PBS (1:1). The left lobes were embedded in OCT and frozen at -80°C or the left lobe was fixed in 10% formaldehyde and embedded in wax. Frozen tissue blocks or wax embedded blocks were sectioned (5–7 μm) and processed for Haematoxylin and Eosin (H&E) and Periodic Acid Schiff (PAS) staining using standard methodology. Quantification of mucus producing area was done using Image J software (Centre for Information Technology, Nation Institute of Health, Bethesda, Maryland). Percentage mucus area/airway was quantified from representative 15–25 equally sized airways.

### Flow cytometry

Flow cytometry was performed using standard methodology. For intracellular cytokine staining of pulmonary lymphocytes, lungs were isolated, digested with Liberase (25 μg/ml) and DNAse I (50 μg/ml) and passed through a 70 μm and a 40 μm strainer to obtain single cell suspensions. Cells were plated in 96 well plates at 2x10^6^ cells/well and stimulated with phorbol 12-myristate 13-acetate (PMA) (50 ng/ml), ionomycin (250 ng/ml) (both Sigma) for 5 hrs. Brefeldin A (5 μg/ml) was added 1 hr after stimulation. Stimulated cells were then washed and stained with surface markers. Cells were subsequently fixed, permeabilized and then stained for intracellular cytokines.

Antibodies used in these experiments included, phycoerythrobilin (PE)- conjugated anti-Siglec-F (clone, E50-2440), PerCPCy5.5- conjugated anti-CD11c (clone, HL3), -CD45.1 (clone, A20), Allophycocyanin (APC)- conjugated anti-Gr-1 (clone, RB6-8C5), -CD11b (clone, M1/70), AlexaFlouro 700- conjugated anti-IFN-γ (clone, XMG1.2), PE-CF594- conjugated anti-RORγt (clone, Q31-378), V450- conjugated anti-IL-4 (clone, 11B11), V500-CD3ε (clone 145-2C11), APC-Cy7-conjugated anti-CD4 (clone RM4-5) were purchased from BD Biosciences. PE-F4/80 (clone, BM8) was purchased from Serotec, PerCP Cy5.5-conjugated anti-IL-17A (clone, TC11-18H10), PE- conjugated anti-GATA3 (clone, TWAJ) and eFlouro 660-conjugated anti-IL-13 (clone, eBio13A) were purchased from eBiosciences. 7AAD or live/death eFluor 450 (clone, 17A2) from eBioscience was used to differentiate dead from live cells. Flow cytometry was performed on Calibur or LSRII instruments (BD Biosciences, San Jose, USA) and analysed with FlowJo 9.4.11 software (Tree Star Inc., Ashland, USA).

### T cell transfers and stimulations

Naive 1-Derβ T cells were isolated from spleens and lymph nodes and labelled with CFSE (Invitrogen). CFSE-labelled un-purified cells (1x10^7^ cells/mouse) were adoptively transferred (i.v.) into recipient mice 2 hrs prior to sensitization, as described in the text. Three days post-sensitization, MLNs were harvested from recipient mice for analysis. The proliferation and expression of transcription factors were analysed with flowcytometry (Fortessa, BD). Percentage division of adoptively transferred CD45.1^+^ CD4^+^ 1derβ T cells was calculated based on the percentage of initial T cells that divided based on CFSE dilution peaks (FlowJo software). To measure %GATA3 and RORγt expressing T cells, MLN single cells were stained for surface CD45.1 and CD4, followed by intracellular detection of GATA3 and RORγt.

For T-cell stimulations, MLN single cell suspensions (2x10^6^ cells/well) were harvested three days post sensitization and stimulated for 3 days with HDM (15 μg/ml). Cytokines were analysed by ELISA (eBioscience), according to manufacturer’s instructions.

### Airway hyperresponsiveness (AHR) measurements

Airway resistance after methacholine challenge was measured as described previously [20] using a Flexivent system (SCIREQ, Montreal, Canada), used according to the manufacturer’s instructions. Mice were anaesthetized with ketamine/xylazine, intubated with an 18G cannula and mechanically ventilated at a frequency of 2.5Hz. Anaesthetized mice were exposed to increasing doses of aerosolized acetyl-methylcholine (methacholine; Sigma-Aldrich, Germany; 0–320 mg/ml for C57BL6 mice and 0–40 mg/ml for Balb/c mice) for 10 seconds per dose, and 15 single-frequency forced oscillation manoeuvres using the single-compartment (“snapshot”) perturbation were performed to determine dynamic airway resistance (R). R is represented as the maximum response for each concentration.

### In situ hybridizations

Mouse CCL5 cDNA was RT-PCR amplified with primers 5’ATGAAGATCTCTGCAGCTGCCC 3’ and 5’CTAGCTCATCTCCAAATAGT 3’. PCR products were ligated to the pGEM-T vector (Promega) and sequenced. The pGEMT- plasmids were then linearized by restriction digest and gene-specific riboprobes were synthesized by in vitro transcription using a Maxiscript SP6/T7 kit (Ambion). Unincorporated nucleotides were removed using RNA Mini Quick Spin Columns (Roche). Paraffin embedded tissue specimens were pre-treated as described [22], following deparaffinization in xylene and rinsing in ethanol. *In situ* hybridization with ^35^S-labeled riboprobes was performed at 50°C overnight as described [22], with 0.1M dithiothreitol included in the hybridization mix. Tissue sections were coated with NTB emulsion (Kodak) and exposed at 10°C for 14 days. The sections were counterstained with Hematoxylin (Vector) and mounted with Permount (Fisher). Images were captured using a Nikon Eclipse E600 microscope, a Nikon DS-Ri1 camera, and Nikon NIS-Elements software.

### Statistical analysis

Statistical significance was determined using Two-tailed, Mann-Whitney test or Two-way ANOVA, followed by Bonferroni post-test analysis for multiple comparisons. All graphs were prepared using GraphPad Prism version 5.0 software (GraphPad, La Jolla, CA). Results are expressed as mean ±SEM or ±SD.

*P* value < 0.05 was considered statistically significant.

## Results

### Microbial ligands act synergistically to promote neutrophilic airway inflammation

**Fig 1:**
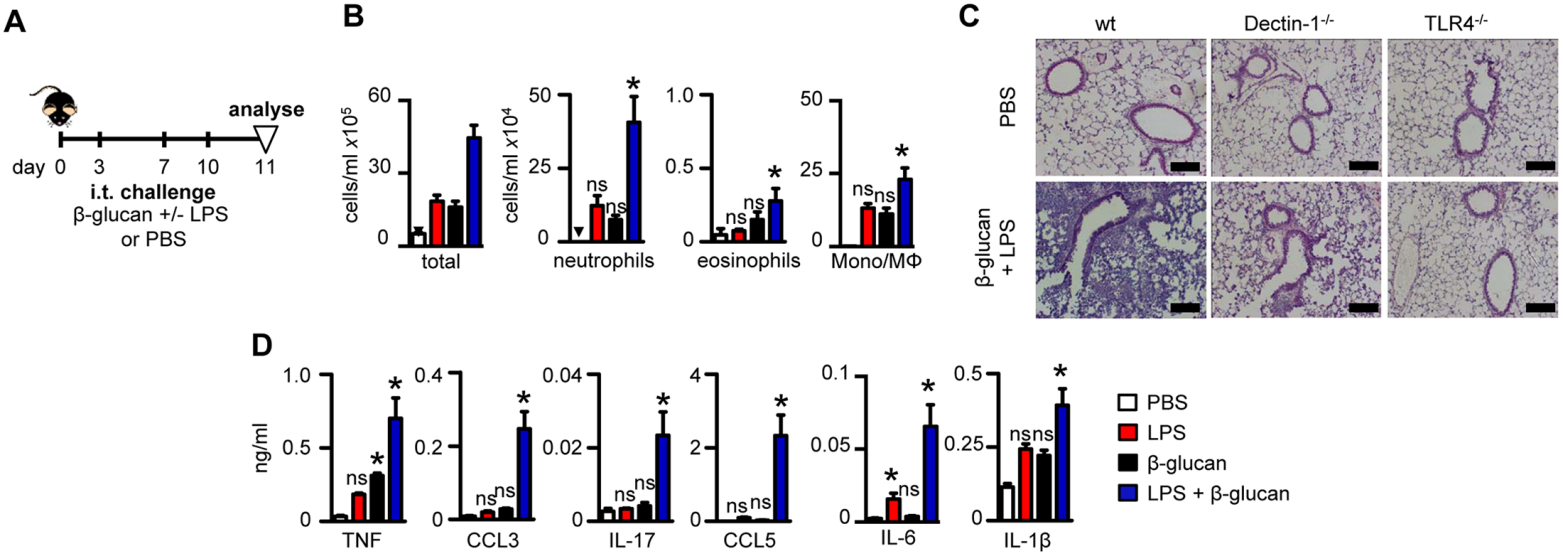
Co-stimulation with β-glucans and LPS elicits robust neutrophilic inflammation. (*A*) Timeline for challenge with β-glucans (1x10^7^ particles), LPS (100 ng), the combination of both agonists, or PBS alone. (B) Total leukocytes, neutrophils (Gr-1^hi^ CD11b^hi^F4/80^lo^), eosinophils (Siglec-F^hi^Gr-1^lo^CD11c^lo^), inflammatory macrophage/monocytes (F4/80^hi^CD11b^hi^Gr-1^lo^) and T cells (CD3^hi^CD4^hi^) in the BALF of C57BL/6 mice following challenge with the various agonists, as indicated. (C) H&E stained lung sections from wild type (wt), Dectin-1^-/-^ or TLR-4^-/-^ mice challenged with PBS or the combination of β-glucan and LPS. Scale bars represent 50 μm. (D) Cytokine levels in the BALF following challenge, as indicated. *p<0.05, n.s., not significant. Shown are the mean ± SEM of pooled data from at least 2 independent experiments (n = 8–10 mice/group).

To determine the effect of costimulatory responses on airway inflammation, we challenged mice intratracheally (i.t.) with highly purified particulate β-glucan and / or LPS and monitored pulmonary cellular recruitment 24 hrs after the last challenge (Fig 1A). While each of the individual ligands induced a low level response, challenge with both agonists resulted in robust neutrophilic inflammation (Fig 1B) which involved the β-glucan receptor, Dectin-1 [23], and TLR-4 (Fig 1C). Low level eosinophilia and influx of inflammatory macrophage/monocytes were also detected upon challenge with β-glucan, either alone or in combination with LPS (Fig 1B). In line with the cellular observations, challenge with β-glucan and LPS induced the synergistic production of several pro-inflammatory cytokines and chemokines, including TNF-α, CCL3 (MIP-1α), IL-17, CCL5 (RANTES), IL-1α, IL-6 and CCL4 (MIP-1β) (Fig 1D and data not shown), which were variably dependent on Dectin-1 and TLR-4 (Fig 1D and S1A Fig). Similar results were obtained in whole lung digests (S1B Fig) or even after a single challenge with these agonists (S2 Fig). Thus, these data show that microbial ligands can act synergistically to induce neutrophilic pulmonary inflammation.

### Microbial ligands promote neutrophilic inflammation during allergic asthma

**Fig 2:**
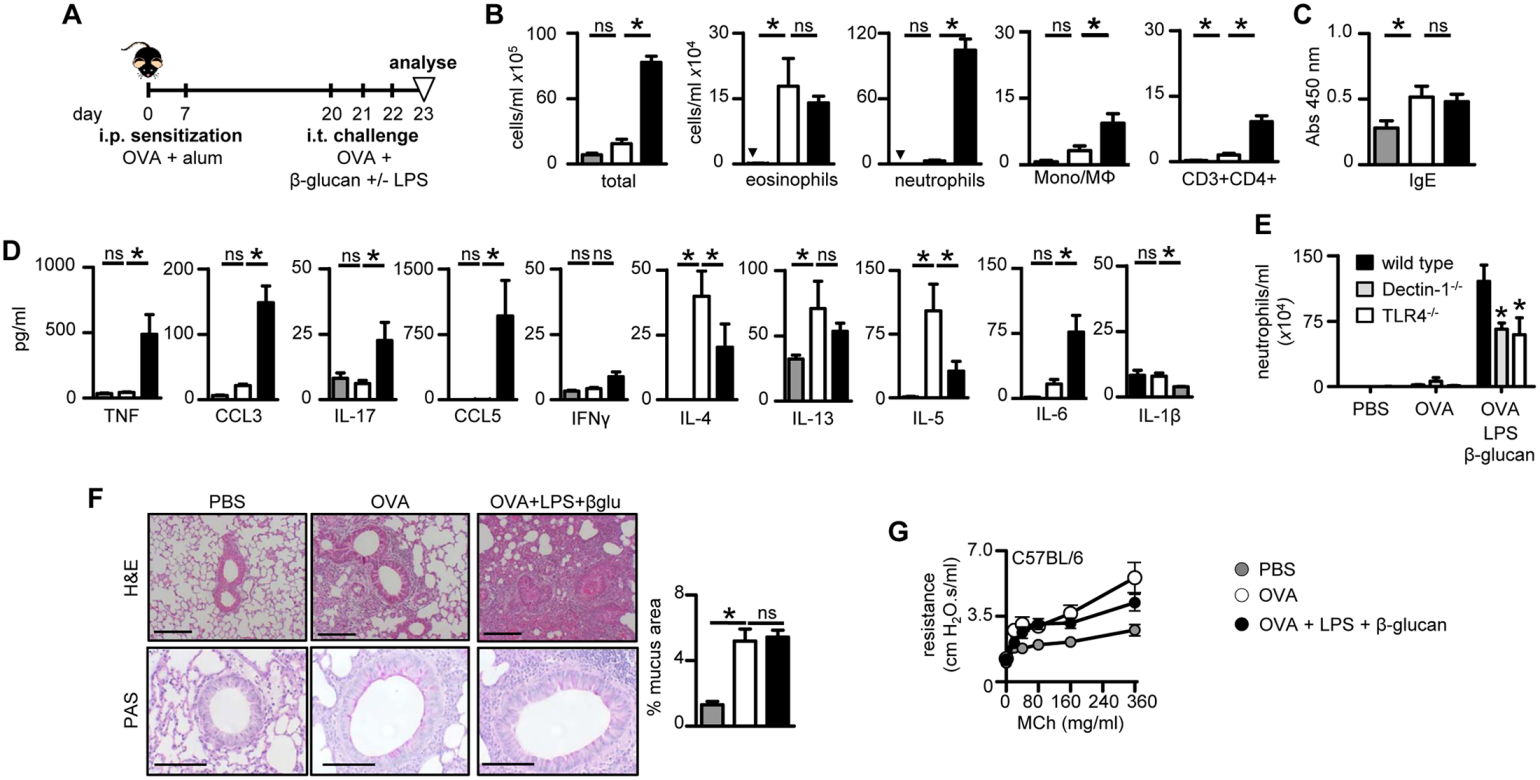
β-Glucan plus LPS promote neutrophilic inflammation in OVA-induced allergic inflammation. (A) Timeline for OVA i.p. sensitizations (25 μg in 1 mg alum) and i.t. challenges (10 μg) with the combination of OVA, β-glucan (1x10^7^ particles) and LPS (100 ng), as indicated. (B) Airway inflammation in challenged C57BL/6 mice showing the number of total leukocytes, eosinophils (Siglec-F^hi^Gr-1^lo^CD11c^lo^), neutrophils (Gr-1^hi^CD11b^hi^F4/80^lo^), inflammatory macrophage/monocytes (F4/80^hi^CD11b^hi^Gr-1^lo^) and T cells (CD3^hi^CD4^hi^). (C) Total serum IgE levels in challenged mice, as indicated. (*D*) Pulmonary cytokine concentrations in BALF of challenged C57BL/6 mice, as indicated. (E) Numbers of recruited neutrophils in the BALF of challenged C57BL/6 wild type, Dectin-1^-/-^ and TLR-4^-/-^ mice, as indicated. (F) H&E and PAS stains of formalin fixed lung sections (left) from C57BL/6 wild type mice, challenged as indicated. Scale bars represent 100 μm (H&E) and 50 μm (PAS). Quantification (right) of mucus producing goblet cell area in PAS stained sections. (G) Airway resistance (R) in intubated C57BL/6 wild type mice exposed to increasing doses of nebulised methacholine (MCh), as indicated. *p<0.05, n.s., not significant. Shown are the mean ± SEM of pooled data from at least 2 independent experiments (n = 14–16 mice/group).

To explore the effect of β-glucans and LPS on allergic pulmonary responses, we first utilized a classic Th2-type eosinophilic allergic model involving sensitization intra-peritoneally (i.p.) of mice with OVA plus Alum. These mice were subsequently challenged i.t. with OVA or OVA along with β-glucans and LPS (Fig 2A). Challenge of sensitized mice with OVA induced robust eosinophilia, as expected [24], which was largely unaffected by the presence or absence of β-glucan and LPS (Fig 2B). Moreover, the administration of these microbial ligands did not substantially alter OVA-specific serum IgE levels (Fig 2C). These responses were unaffected by Dectin-1 or TLR-4-deficiency (S3A Fig), consistent with previous observations [24].

In contrast, inclusion of β-glucan and LPS during OVA challenge induced a robust adjunctive neutrophilic inflammation (Fig 2B). This was characterized by an inflammatory Th17-type cytokine profile, and a suppressive effect on Th2-type cytokines, including IL-4 and IL-5 (Fig 2D). Robust neutrophilic responses involved both Dectin-1 and TLR-4, as neutrophil recruitment was reduced in mice lacking either of these receptors (Fig 2E).

Histological analysis confirmed these observations, with the highest levels of pulmonary inflammation occurring following challenge with the combination of β-glucan and LPS (Fig 2F). This inflammation was not only restricted to perivascular and peribronchiolar environments, it also infiltrated the alveoli space, suggesting a profound effect on lung inflammation. This allergic model also led to increased goblet cell hyperplasia, as expected [25], which was unaffected by presence of β-glucans and LPS (Fig 2C). We also did not detect any substantial alterations in AHR following challenge with the bronchoconstrictor methacholine in either C57BL/6 or Balb/c mice (Fig 2G and S3B Fig).

In the mouse, the route of sensitization and the presence or absence of alum adjuvant affects the nature of the subsequent allergic response [7, 13, 26]. Thus, we investigated whether sensitizing through the airway with combinations of LPS and β-glucan would alter pulmonary inflammatory responses (S4A Fig). Mice sensitized through the airway and then challenged with OVA in the absence of alum adjuvant showed no response, as expected [13]. In contrast, mice sensitized through the airway with Ova and both agonists developed a robust neutrophilic inflammatory response with low-level eosinophilia upon rechallenge with OVA alone (S4B Fig). Inflammation was mainly characterized by enhanced production of CCL5 and other chemokines, but interestingly not IL-17 (S4C Fig). As before, neutrophilic inflammation in this model involved Dectin-1 and TLR-4 (S4D Fig). These responses were induced following antigen administration, as inflammation at these later time points was resolved in mice sensitized only with LPS and β-glucan (S5 Fig). Thus, these data show that the combination of LPS and β-glucan acts as an adjuvant during airway sensitization to harmless protein allergens, promoting robust neutrophilic recall responses to inhaled antigens.

### Microbial ligands promote Th2 and Th17 allergic airway inflammation in response to house dust mite

**Fig 3:**
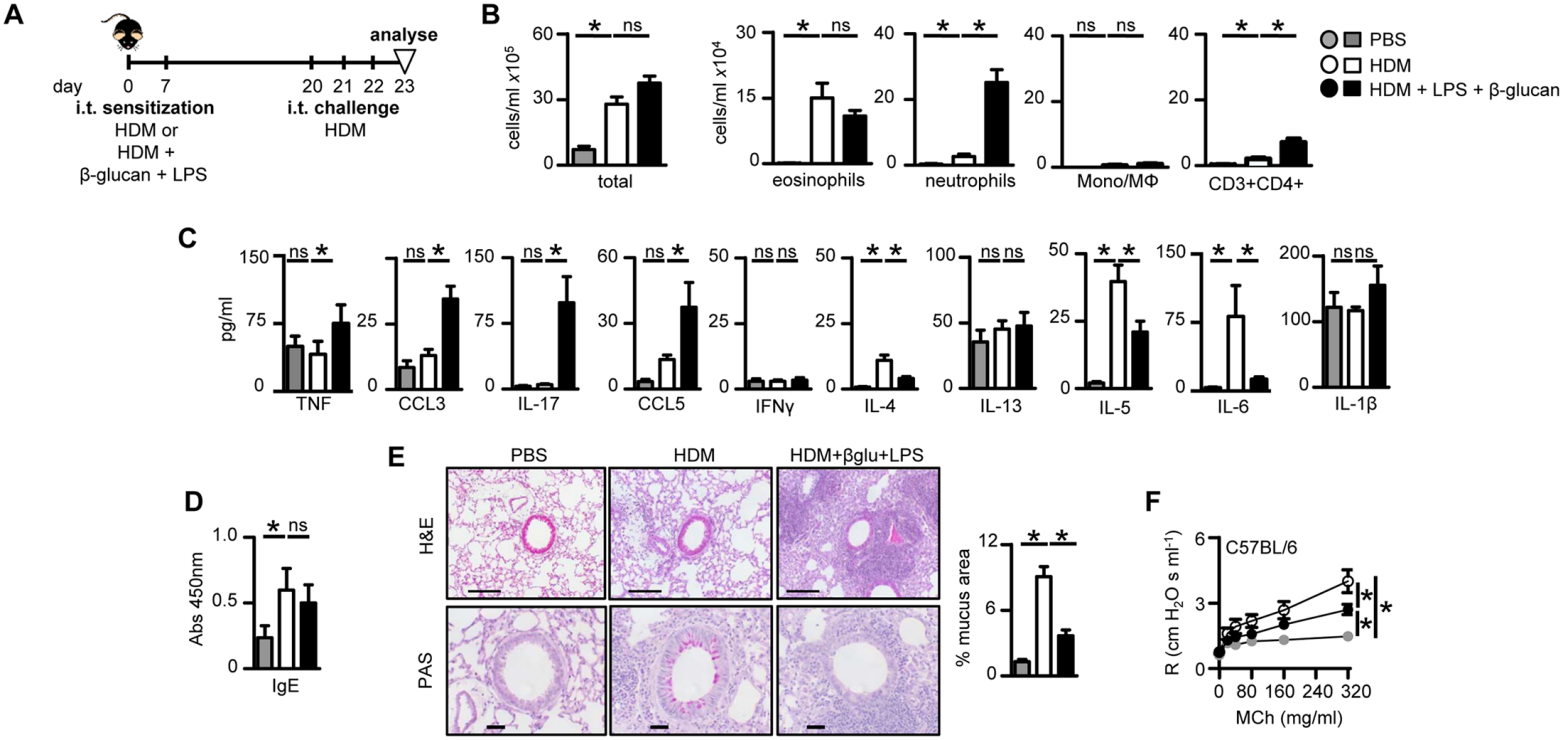
β-Glucan and LPS modulate HDM induced allergic airway inflammation. (A) Timeline for HDM sensitization and challenge (10 μg) in C57BL/6 wild type mice with HDM or HDM plus β-glucan (1x10^7^ particles) and LPS (100 ng), as indicated. (B) Airway inflammation in challenged C57BL/6 mice showing the number of total leukocytes, eosinophils (Siglec-F^hi^Gr-1^lo^CD11c^lo^), neutrophils (Gr-1^hi^CD11b^hi^F4/80^lo^), inflammatory macrophage/monocytes (F4/80^hi^CD11b^hi^Gr-1^lo^) and T cells (CD3^hi^CD4^hi^) in the BALF. (C) Pulmonary cytokine concentrations in BALF of challenged mice, as indicated. (D) HDM-specific serum IgE levels in challenged mice, as indicated. (E) H&E and PAS stains (left) of formalin fixed lung sections from mice challenged as indicated. Scale bars represent 100 μm (H&E) and 50 μm (PAS). Quantification (right) of mucus producing goblet cells in PAS stained sections. (F) Airway resistance (R) in intubated C57BL/6 wild type mice exposed to increasing doses of nebulised methacholine (MCh), as indicated. *p<0.05, n.s., not significant. Shown (b–f) are the mean ± SEM of pooled data from at least two independent experiments (n = 14–16 mice/group).

To translate our observations to a clinically more relevant allergen, we examined the effects of LPS and β-glucan on the airway inflammation induced by house dust mite (HDM) crude extracts. We used crude extracts that were low in endotoxin levels (see Materials and Methods) compared those used in previous experiments [7, 27]. We used intra-tracheal sensitization protocol with HDM without adjuvants like alum, as this is the most representative route for studying pulmonary allergic responses [7]. For these experiments mice were sensitized i.t. with HDM extracts plus the microbial ligands and then challenged with HDM alone two weeks later (Fig 3A). As expected [7, 28], sensitization and challenge with HDM alone induced pronounced eosinophilic airway inflammation (Fig 3B), characterized by a Th2-type cytokine profile (Fig 3C). We also detected increased HDM-specific IgE (Fig 3D) and goblet cell metaplasia (Fig 3E), as well as exacerbated AHR (Fig 3F), consistent with the induction of a Th2-type allergic response.

The addition of LPS and β-glucan during sensitization with HDM induced robust adjunctive neutrophilic inflammation upon HDM challenge (Fig 3B), similar to what we had observed in the OVA challenge model (Fig 2 and S4 Fig). We could also show that adjunctive neutrophilic inflammation was similar in both BALF and whole lung digest (S6A Fig). These responses were substantially reduced in mice lacking Dectin-1 or TLR-4 (S6B Fig). The neutrophilic inflammation was accompanied by a strong pro-inflammatory Th17-type cytokine response upon HDM challenge, characterized by increased levels of TNF-α, CCL3, IL-17, CCL5 and several other cytokines (Fig 3C and data not shown). There was no effect on the IgE response (Fig 3D), but there were reduction in the level of IL-4 and IL-5 (Fig 3C), goblet cell metaplasia (Fig 3E) and AHR following methacholine challenge (Fig 3F). Interestingly, similar results were obtained using Balb/c mice when compared to C57BL/6 despite known strain differences and Balb/c being a preferred strain for Th2-allergic models (S7 Fig and data not shown).

### Sensitization with microbial ligands alters T-cell polarisation

**Fig 4:**
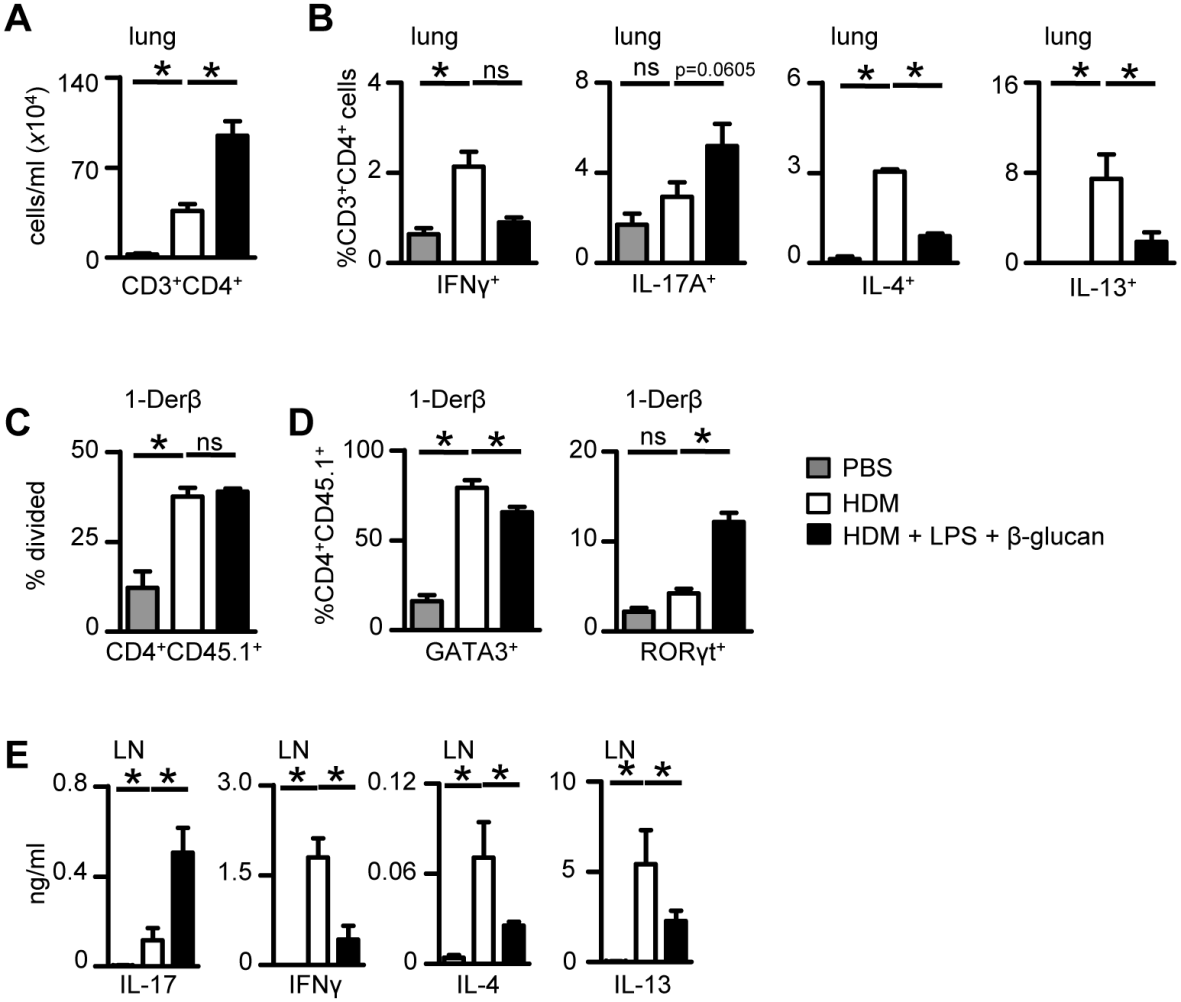
β-Glucan and LPS influence T-cell responses to HDM. (A) Numbers of CD4^+^ T- cells in the BALF in WT (C57BL6) mice 24 hr post-last challenge with HDM alone or together with LPS and β-glucans as described in Fig 3. (B) Intracellular staining for cytokines in CD3^+^CD4^+^ lymphocytes isolated from the lungs of mice treated as in (A). (C) Proliferation (% division) of 1-Derβ T cells (CD3^+^CD4^+^CD45.1^+^) in the MLN of mice three days after sensitization with HDM alone or HDM plus LPS and β-glucan. (D) GATA3 and RORγt expression in adoptively transferred 1-Derβ specific T cells (CD4^+^CD45.1^+^) in the MLN of mice three days after sensitization with HDM alone or HDM plus LPS and β-glucan. (E) Cytokine concentrations in MLN cell suspensions, isolated from mice three days after sensitisation with HDM alone or HDM plus LPS and β-glucan. MLN cells were restimulated *ex vivo* with HDM for 3 days. *p<0.05, n.s., not significant. Shown are the mean ± SD from one representative experiment of 2 experiments (n = 4–5 mice/group).

Given the remarkable ability of β-glucan and LPS to modulate allergic airway responses, we next explored their effects on T-cell immunity. Sensitization with these agonists increased the number of αβ CD4^+^ T-cells in the lung following HDM challenge (Fig 4A), which we could show to produce IFN-γ, IL-17, IL-4 and IL-13 (Fig 4B). Notably, β-glucan and LPS repressed cells producing IL-4, IL-13 and IFN-γ and tended to increase the number of T–cells producing IL-17.

To gain further insights, we made use of our recently generated T cell receptor (TCR) transgenic (Tg) mouse (1-Derβ Tg) that recognises an immunodominant peptide from the HDM-derived allergen, Derp-1 [18]. We could show that adoptively transferred 1-Derβ T cells proliferated in mice sensitized with HDM or HDM plus β-glucan and LPS (Fig 4C and S8A Fig). These cells did not proliferate in mice sensitized with β-glucan and LPS alone (S8B Fig). In mice sensitized with HDM, these cells expressed high levels of GATA3, which was reduced in cells isolated from mice sensitized in the presence of the microbial agonists (Fig 4D and S8A Fig). There were high levels of Th2-associated cytokines produced following *in vitro* re-stimulation of MLN cells from mice sensitized with HDM, including IL-4 and IL-13 (Fig 4E), which were significantly repressed in cells from mice in which the microbial agonists had been included during sensitization (Fig 4E). Sensitization with both LPS and β-glucan induced higher levels of RORγt expression in adoptively transferred 1-Derβ T-cells and IL-17 in restimulated MLN cells *in vitro* (Fig 4D and 4E and S8A Fig). Mice challenged with HDM also induced IFN-γ-producing T-cells, whose level were reduced in cells from mice sensitized with microbial agonists (Fig 4E). Thus, these data demonstrate that the microbial ligands substantially alter T-cell polarization during allergic inflammation, promoting a shift towards Th17-type responses.

### Sensitization with β-glucan plus LPS drives steroid refractory asthma

**Fig 5:**
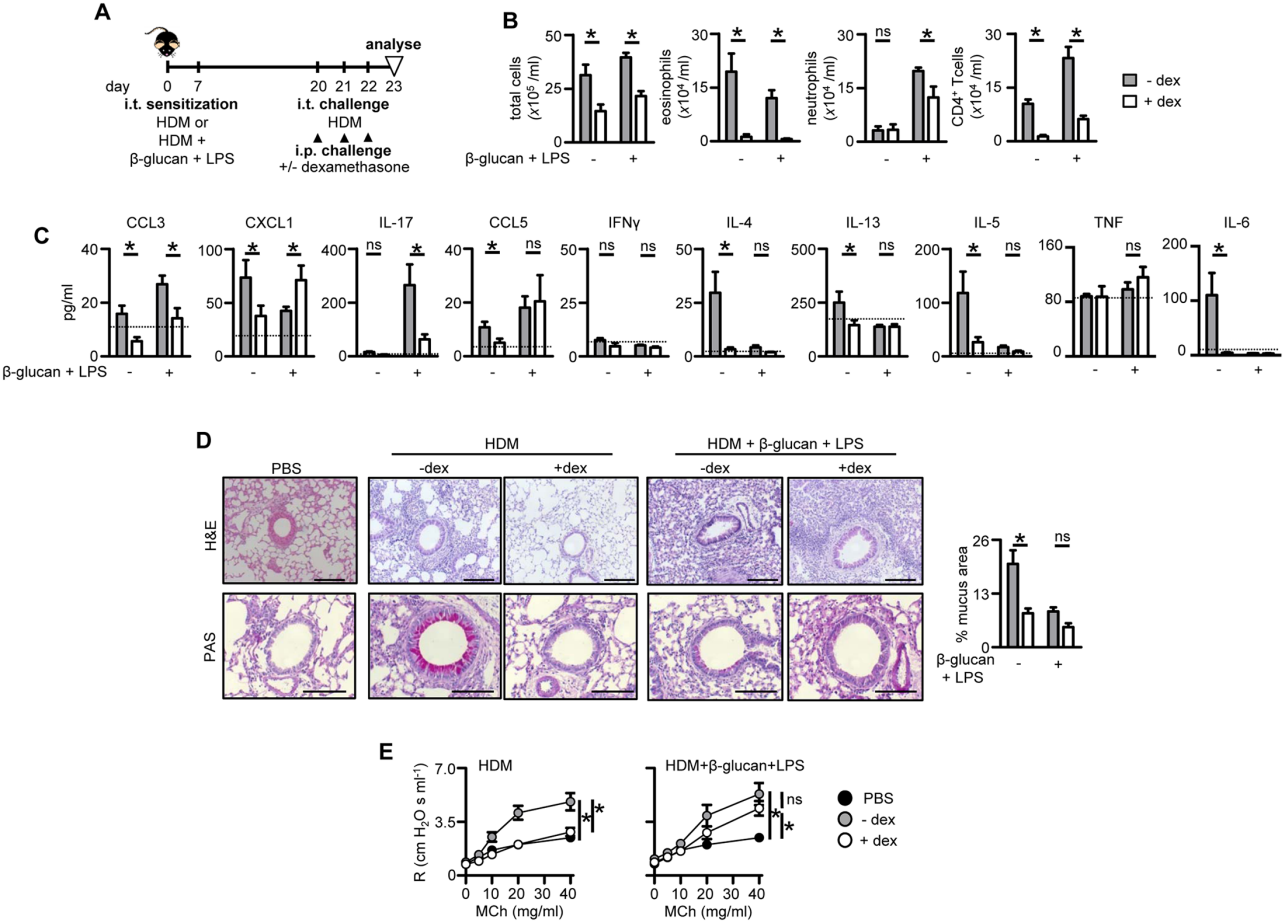
β-Glucan plus LPS promote corticosteroid resistant airway inflammation. (A) Timeline for HDM sensitization and challenge, as in Fig 4, with and without i.p. dexamethasone (dex). (B) Airway inflammation in challenged and dex-treated C57BL/6 mice showing the number of total leukocytes), eosinophils (Siglec-F^hi^Gr-1^lo^CD11c^lo^), neutrophils (Gr-1^hi^CD11b^hi^F4/80^lo^) and T- cells (CD3^+^CD4^+^) in the BALF. (C) Pulmonary cytokine concentrations in BALF of challenged and treated mice, as indicated. The dotted line represents levels found in control (PBS) mice. (D) H&E and PAS stains (right) of formalin fixed lung sections from mice challenged as indicated. Scale bars represent 100 μm (H&E) and 50 μm (PAS). Quantification (left) of mucus producing goblet cell area in PAS stained sections. (E) Airway resistance (R) in intubated challenged and treated Balb/C wild type mice exposed to increasing doses of nebulised methacholine (MCh), as indicated. *p<0.05, n.s., not significant. Shown are the mean ± SEM of pooled data from two independent experiments (n = 8–10 mice/group).

In human asthma, neutrophilic or mixed granulocytic inflammation is generally associated with severe disease which is resistant to standard treatment with corticosteroids but the underlying mechanisms driving these responses are unclear [4, 29]. We therefore explored the possibility that the neutrophilic inflammation initiated following sensitization with HDM, β-glucan and LPS would be resistant to corticosteroid treatment. For these experiments, sensitized Balb/C animals were treated with high-dose dexamethasone during challenge with HDM (Fig 5A). Balb/C mice were chosen for these experiments as they presented with more robust AHR responses following methacholine challenge, than did C57BL/6 mice. As we had observed earlier, sensitization of mice with HDM and LPS plus β-glucan induced robust adjunctive neutrophilia, while the eosinophilic response was largely unaltered when compared to mice sensitized with HDM alone (Fig 5B). Treatment with dexamethasone reduced total cellular inflammation and substantially reduced the numbers of eosinophils and T-cells recruited to the lung (Fig 5B), correlating with reduced Th2-type cytokines, including IL-4, IL-5 and IL-13 (Fig 5C).

In contrast, neutrophil recruitment in mice sensitized with HDM and LPS plus β-glucan was only slightly affected by dexamethasone treatment (Fig 5B), despite reduced levels of IL-17 and other associated cytokines, such as IL-6 (Fig 5C). However, in these animals, the production of CCL5 was unaffected by corticosteroid treatment while the levels of KC (CXCL1) were increased (Fig 5C). Strikingly, histological inflammation (Fig 5D) as well as airway hyper-responsiveness (Fig 5E) were not affected by corticosteroid treatment in mice sensitized with HDM and β-glucan plus LPS, whereas they were substantially reduced in mice sensitized with HDM alone. Thus, these data show that sensitization to allergens in the presence of these microbial ligands drives the development of steroid-resistant neutrophilic asthma.

## Discussion

The coordinated recognition of different microbial components, particularly those recognised by TLRs and CLRs, can substantially alter the resultant innate and adaptive immune responses [15–17, 30]. Agonists of these receptors, including LPS and β-glucan, are ubiquitous in the environment and have been linked to the development of allergy and asthma [11, 31]. We discovered that the combination of LPS and β-glucan profoundly affects both allergic and non-allergic pulmonary inflammatory responses in a variety of mouse models. Irrespective of the model tested, we found that the combination of these agonists promoted robust neutrophilic inflammation, and involved Dectin-1 and TLR-4. Notably, and reminiscent of patients with severe asthma [32], pulmonary responses in our mouse models were steroid refractory when LPS and β-glucan were included during allergen sensitization. Thus these observations provide new insights into the mechanisms by which microbial agonists can influence the development of allergic and non-allergic pulmonary inflammation, and the etiology of steroid refractory disease.

While the consequences of LPS administration on pulmonary inflammation are well characterised [12, 13, 24, 33], the effects of β-glucan are less clear. These carbohydrates can be inhaled in the form of spores, fungal fragments and from other dust and have been proposed to be causally related to the development of asthma, particularly in indoor and occupational environments [11, 34]. Consequently, there have been several studies exploring the effects of β-glucans on allergic and non-allergic respiratory responses, but the data are inconsistent. Several epidemiological studies, for example, suggest that β-glucans can have detrimental effects; however, such associations have not been found in all cases [9]. Furthermore, there have been contradictory results from experimental challenge studies that been performed in both humans and laboratory animals (see [14, 35] for examples), which are likely to have stemmed from the use of different types and purity of β-glucan. We now know, for example, that solubilised forms of β-glucans lack stimulatory activity [36, 37]. In contrast, particulate glucans induce numerous cellular responses, including cytokine and chemokine production, and they promote the development of innate memory and adaptive immunity [37–39].

The combination of LPS and β-glucan, on the other hand, led to robust neutrophilic inflammation in all models tested. In most cases, this was associated with higher levels of IL-17, a cytokine which induces neutrophil recruitment into the lungs and has been linked to severe asthma in both humans and rodent models [40–44]. In the HDM model, the levels of this cytokine correlated with increased numbers of RORγt^+^ IL-17 producing T-cells in the lungs and lymph nodes. These cells are known to be induced by microbial agonists following allergic sensitization through the airways and have been linked to steroid resistance [13, 42, 45, 46]. Notably, corticosteroid treatment reduced IL-17 levels, as occurred in other Th17-biased mouse models [27] and which has also been observed in severe asthma patients following steroid treatment [47]. LPS and β-glucan could also sensitize mice to OVA, priming neutrophilic recall responses which did not involve IL-17. Rather these responses were associated with several other inflammatory mediators including IL-6, CCL3 and CCL5.

In fact, in all the models tested, CCL5 was the only cytokine which closely correlated with the neutrophilic responses. We could detect this chemokine in multiple cell types in lung tissue following challenge, as revealed by *in situ* hybridization (S9 Fig). The distribution of producer cells was diffuse and in perivascular and peribronchiolar microenvironments with small subsets of cells expressing high levels of CCL5 mRNA. Unlike IL-17, expression of CCL5 was unaffected following corticosteroid treatment. High levels of CCL5 are present in patients with severe disease [48], and although traditionally linked to eosinophil chemotaxis, CCL5 also promotes neutrophil recruitment into the lung during allergic inflammation [49]. Thus our data suggest that CCL5, as opposed to IL-17, may be one of the primary drivers for the neutrophilic responses observed in our models. Despite considerable effort, however, we have been unable to demonstrate this conclusively using antibody depletion with reagents that are commercially available.

Our model using LPS and β-glucan provides important insights and new tools to explore the contribution of environmental factors underlying the development of corticosteroid resistant asthma. Indeed, the understanding of the pathophysiological mechanisms underlying non-classical disease endotypes, particularly severe asthma that often occurs with chronic rhinosinusitis and colonization of the upper airways with fungi, has been hampered by a lack of good models [50]. Current models are complex and cumbersome, involving overexpression of polarizing transcription factors, adoptive transfer of *in vitro* polarized cells, or chronic challenges [27, 42, 45]. Our reductionist approach using purified agonists also demonstrates that studying individual microbial components in isolation does not reveal the complexity of real life allergens where important combinatorial effects come into play. This is particularly relevant for β-glucans, whose effects with other agonists like LPS not only promotes respiratory inflammation but may underlie the well-described association of fungi with severe asthma [51]. Indeed, in mouse models of mould-induced asthma, β-glucans exposed on these organisms were found to induce Th17 responses and neutrophilic inflammation in a Dectin-1-dependent fashion [52–54]. Interestingly, in a pulmonary model investigating inflammation following administration of heat killed *Candida albicans* and *Pneudomonas areginosa*, also induced neutrophilic inflammation [55]. Our study using single components further expands our understanding on the influence of costimulation in the development of pulmonary inflammation, and highlights the importance of using single agonists in the understanding of pulmonary inflammation, which otherwise would be difficult to dissect using whole organisms.

In conclusion, we have discovered that combinations of commonly encountered microbial components drive steroid-refractory asthma and present key new insights into the etiology of this severe, life-threatening, disease.

## Supporting Information

S1 Fig Synergistic cytokine production requires Dectin-1 and TLR-4. (A) Cytokine levels in the BALF of C57BL6 wild type, Dectin-1^-/-^ and TLR-4^-/-^ mice chronically challenged with β-glucans plus LPS as in Fig 1A. (B) Number of neutrophils (Gr-1^hi^CD11b^hi^F4/80^lo^), inflammatory macrophage/monocytes (F4/80^hi^CD11b^hi^Gr-1^lo^) and eosinophils (Siglec-F^hi^Gr-1^lo^CD11c^lo^) in whole lung digests from animals treated as in Fig 1A. *p<0.05, n.s., not significant. Shown are the mean ± SEM of pooled data from two independent experiments (n = 8–10 mice/group).
(PDF)

S2 Fig A single challenge with β-glucan plus LPS induces neutrophilic airway. (A) Timeline for challenge with β-glucan (1x10^7^ particles), LPS (100 ng), the combination of both agonists, or PBS alone. (B) Numbers of neutrophils (Gr-1^hi^ CD11b^hi^F4/80^lo^, left) and eosinophils (Siglec-F^hi^Gr-1^lo^CD11c^lo^, right) in the BALF of C57BL/6 mice following challenge with the various agonists, as indicated. (C) Numbers of neutrophils in the BALF of wild type, Dectin-1^-/-^ and TLR-4^-/-^ mice following challenge with LPS plus β-glucan. *p<0.05, n.s., not significant. Shown are the mean ± SEM of pooled data from at least two independent experiments (n = 7–8 mice/group).
(PDF)

S3 Fig Eosinophilic inflammation develops independently of Dectin-1 and TLR-4 signalling. (A) Numbers of eosinophils (Siglec-F^hi^Gr-1^lo^CD11c^lo^) in the BALF of wild type (C57BL6), Dectin-1^-/-^ and TLR-4^-/-^ mice sensitized and challenged as in Fig 2. (B) Airway resistance (R) in intubated Balb/C mice wild type mice sensitized and challenged as in Fig 2 and exposed to increasing doses of nebulised methacholine (MCh), as indicated. Shown are the mean ± SEM of pooled data from two independent experiments (n = 8–10 mice/group).
(PDF)

S4 Fig β-Glucan plus LPS sensitize mice to OVA, promoting neutrophilic recall responses. (A) Timeline for OVA sensitization and challenge (10 μg) in C57BL/6 wild type mice with or without β-glucan (1x10^7^ particles) and LPS (100 ng), as indicated. (B) Airway inflammation in challenged C57BL/6 mice showing the number of total leukocytes, eosinophils (Siglec-F^hi^Gr-1^lo^CD11c^lo^), and neutrophils (Gr-1^hi^CD11b^hi^F4/80^lo^) in the BALF. (C) Pulmonary cytokine concentrations in BALF of challenged mice, as indicated. (D) Number of neutrophils (Gr-1^hi^CD11b^hi^F4/80^lo^) in the BALF wild type C57BL/6, Dectin-1^-/-^ and TLR-4^-/-^ mice sensitized and challenged as in A. Shown are the mean ± SEM of pooled data from three independent experiments (n = 10–12 mice/group). *p<0.05, n.s., not significant.
(PDF)

S5 Fig Reduction in inflammation over time following sensitization of mice with β-glucan plus LPS. (A) Timeline for sensitization and analysis in C57BL/6 wild type mice with β-glucan (1x10^7^ particles) and LPS (100 ng), as indicated. (B) Airway inflammation in challenged C57BL/6 mice showing the number of total leukocytes, eosinophils (Siglec-F^hi^Gr-1^lo^CD11c^lo^), and neutrophils (Gr-1^hi^CD11b^hi^F4/80^lo^) in the BALF. Shown are the mean ± SEM of pooled data from two independent experiments (n = 4–8 mice/group).
(PDF)

S6 Fig Neutrophilic inflammation induced following sensitization with HDM and β-glucan plus LPS is similar in whole lung and is dependent on Dectin-1 and TLR-4 signalling. (A) Number of neutrophils (Gr-1^hi^CD11b^hi^F4/80^lo^), inflammatory macrophage/monocytes (F4/80^hi^CD11b^hi^Gr-1^lo^) and T cells (CD3^hi^CD4^hi^) in whole lungs of mice challenged as in Fig 3. (B) Airway inflammation in challenged wild type C57BL/6, Dectin-1^-/-^ and TLR-4^-/-^ mice sensitized and challenged as in Fig 3, showing the number of neutrophils (Gr-1^hi^CD11b^hi^F4/80^lo^) in the BALF. Shown are the mean ± SEM of pooled data from two independent experiments (n = 8–10 mice/group).
(PDF)

S7 Fig The effects of β-glucan plus LPS on HDM-induced pulmonary inflammation is not mouse strain dependent. (A) Timeline for HDM sensitization and challenge (10 μg) in Balb/c wild type mice with HDM alone (10 μg) or with the combination of β-glucan (1x10^7^ particles) plus LPS (100 ng), as indicated. (B) Airway inflammation in challenged Balb/c mice showing the number of total leukocytes (left), eosinophils (Siglec-F^hi^Gr-1^lo^CD11c^lo^, middle), and neutrophils (Gr-1^hi^CD11b^hi^F4/80^lo^,right) in the BALF. (C) H&E and PAS stains of formalin fixed lung sections (right) from mice challenged as indicated. Scale bars represent 100 μm (H&E) and 50 μm (PAS). (D) Airway resistance (R) in intubated Balb/c wild type mice exposed to increasing doses of nebulised methacholine (MCh), as indicated. *p<0.05, n.s., not significant. Shown are the mean ± SEM of pooled data from two independent experiments (n = 7–8 mice/group).
(PDF)

S8 Fig Sensitization with HDM, β-glucan and LPS activate and polarise HDM-specific T cells (1-Derβ TCR T cells). (A) FACS plots representing CD4^+^ T cell proliferation (CFSE dilution frequency), GATA3 (CD45.1^hi^GATA3^+^) and RORγt (CD45.1^hi^RORγt^+^) expression in adoptively transferred 1-Derβ T cells (CD4^+^CD45.1^+^) isolated from the MLN of mice challenged as in Fig 4. (B) FACS plots showing CD4^+^ T cell proliferation (CFSE dilution frequency) following sensitization with HDM, β-glucan and LPS or β-glucan and LPS alone, in adoptively transferred 1-Derβ T cells (CD4^+^CD45.1^+^) isolated from the MLN of mice challenged as in Fig 4.
(PDF)

S9 Fig *In situ* hybridization for CCL5 mRNA in mouse lung tissues. A CCL5-specific riboprobe was hybridized *in situ* to localize producer cells and signal is evident by collections of black silver grains over individual cells. Shown are micrographs from C57BL/6 wild type mice sensitized with HDM, β-glucan and LPS (100 ng) treated with or without dexamethasone, as indicated, at x100, x200, and x400 magnifications following *in situ* hybridization with an antisense riboprobe. A control is shown to the right, for which the tissue was probed with a CCL5 sense riboprobe, to show the level of background signal.
(PDF)
