# Correction: Microbial Ligand Costimulation Drives Neutrophilic Steroid-Refractory Asthma

**DOI:** 10.1371/journal.pone.0137945

**Published:** 2015-09-08

**Authors:** Sabelo Hadebe, Frank Kirstein, Kaat Fierens, Kong Chen, Rebecca A. Drummond, Simon Vautier, Sara Sajaniemi, Graeme Murray, David L. Williams, Pierre Redelinghuys, Todd A. Reinhart, Beth A. Fallert Junecko, Jay K. Kolls, Bart N. Lambrecht, Frank Brombacher, Gordon D. Brown

There is an error in Affiliation 1 for authors Sabelo Hadebe, Rebecca A. Drummond, Simon Vautier, Sara Sajaniemi, Pierre Redelinghuys, and Gordon D. Brown. Affiliation 1 should be: Aberdeen Fungal Group, Infection, Immunity and Inflammation Programme, University of Aberdeen, United Kingdom.
