# Supplementary figures and images for: Microbial Ligand Costimulation Drives Neutrophilic Steroid-Refractory Asthma
Source: PLoS One. 2015 Aug 11;10(8):e0134219. doi: 10.1371/journal.pone.0134219 (PMC4532492; doi:10.1371/journal.pone.0134219)

**A**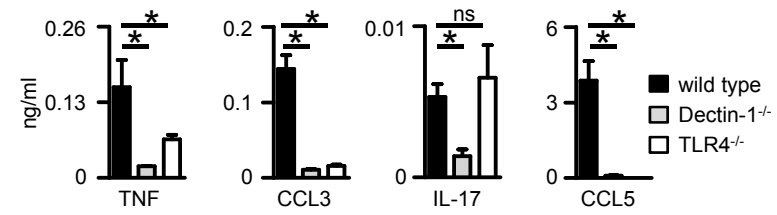**B**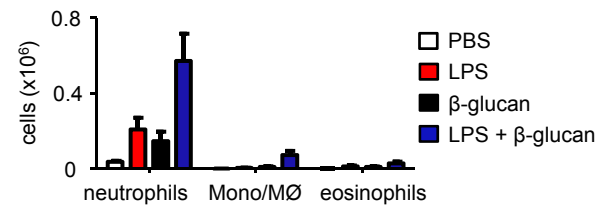

Supplement: S1 Fig — (A) Cytokine levels in the BALF of C57BL6 wild type, Dectin-1-/- and TLR-4-/- mice chronically challenged with β-glucans plus LPS as in Fig 1A. (B) Number of neutrophils (Gr-1hiCD11bhiF4/80lo), inflammatory macrophage/monocytes (F4/80hiCD11bhiGr-1lo) and eosinophils (Siglec-FhiGr-1loCD11clo) in whole lung digests from animals treated as in Fig 1A. *p<0.05, n.s., not significant. Shown are the mean ± SEM of pooled data from two independent experiments (n = 8–10 mice/group). (PDF) [file pone.0134219.s001.pdf]

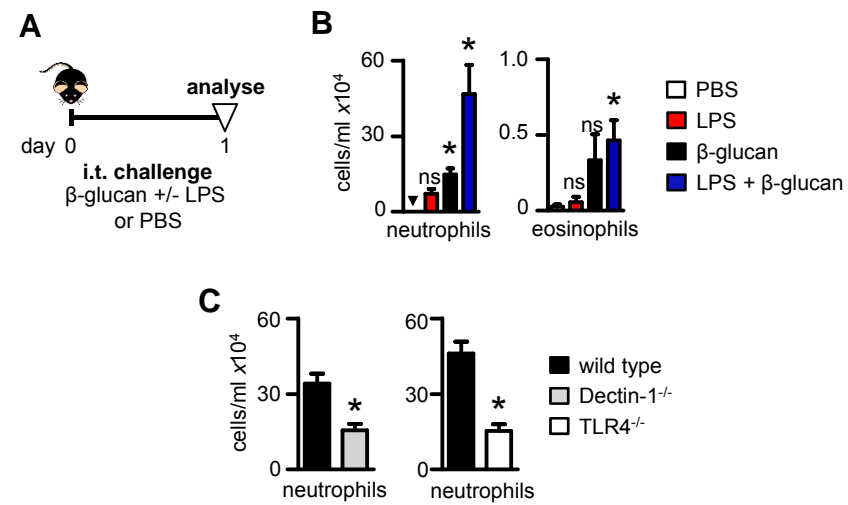

Supplement: S2 Fig — (A) Timeline for challenge with β-glucan (1x10^7^ particles), LPS (100 ng), the combination of both agonists, or PBS alone. (B) Numbers of neutrophils (Gr-1hi CD11bhiF4/80lo, left) and eosinophils (Siglec-FhiGr-1loCD11clo, right) in the BALF of C57BL/6 mice following challenge with the various agonists, as indicated. (C) Numbers of neutrophils in the BALF of wild type, Dectin-1-/- and TLR-4-/- mice following challenge with LPS plus β-glucan. *p<0.05, n.s., not significant. Shown are the mean ± SEM of pooled data from at least two independent experiments (n = 7–8 mice/group). (PDF) [file pone.0134219.s002.pdf]

**A**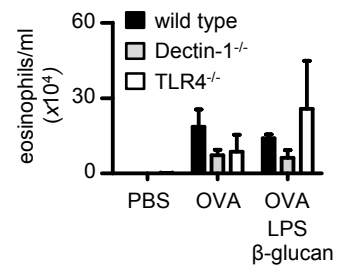**B**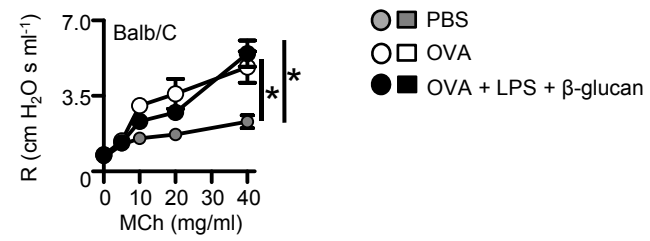

Supplement: S3 Fig — (A) Numbers of eosinophils (Siglec-FhiGr-1loCD11clo) in the BALF of wild type (C57BL6), Dectin-1-/- and TLR-4-/- mice sensitized and challenged as in Fig 2. (B) Airway resistance (R) in intubated Balb/C mice wild type mice sensitized and challenged as in Fig 2 and exposed to increasing doses of nebulised methacholine (MCh), as indicated. Shown are the mean ± SEM of pooled data from two independent experiments (n = 8–10 mice/group). (PDF) [file pone.0134219.s003.pdf]

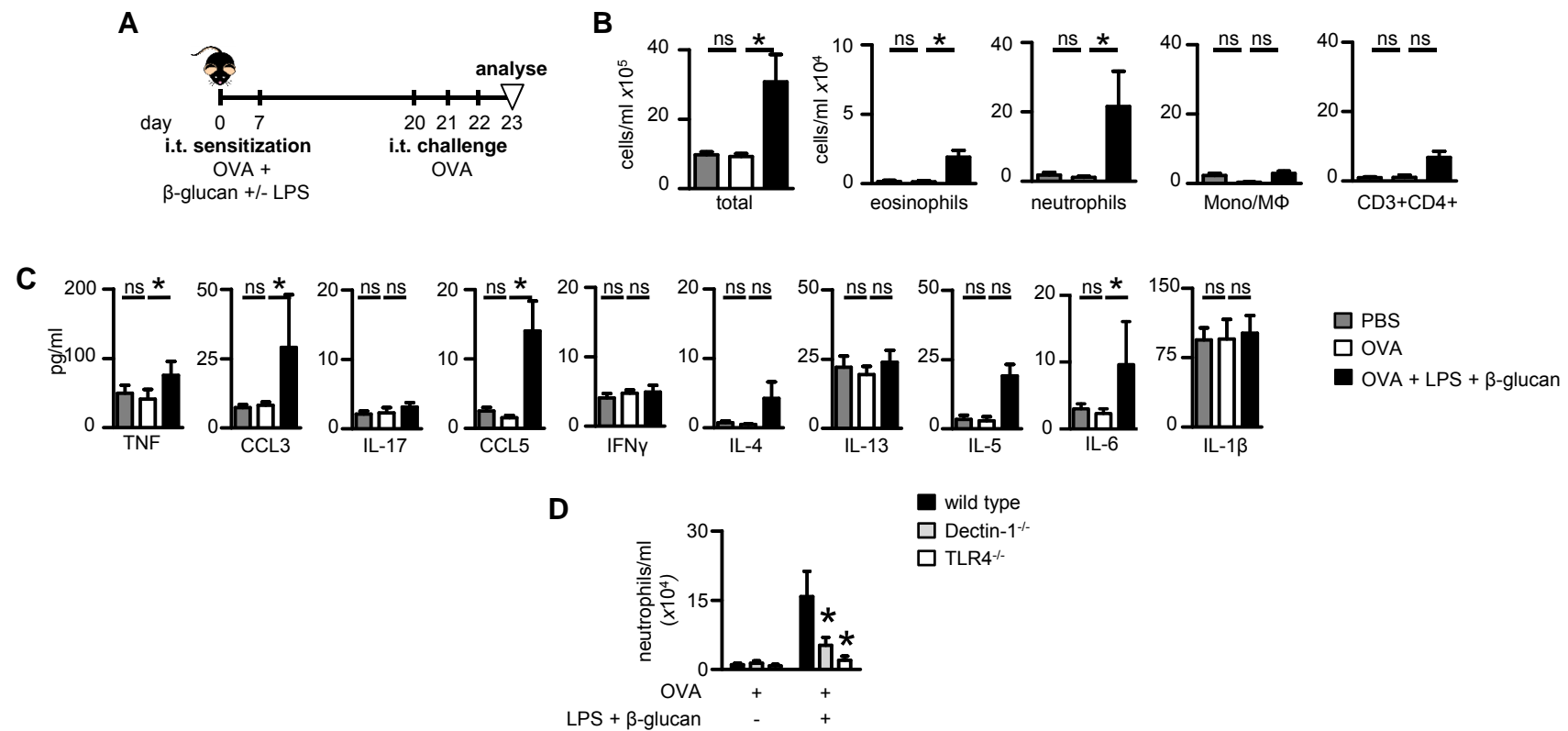

Supplement: S4 Fig — (A) Timeline for OVA sensitization and challenge (10 μg) in C57BL/6 wild type mice with or without β-glucan (1x10^7^ particles) and LPS (100 ng), as indicated. (B) Airway inflammation in challenged C57BL/6 mice showing the number of total leukocytes, eosinophils (Siglec-FhiGr-1loCD11clo), and neutrophils (Gr-1hiCD11bhiF4/80lo) in the BALF. (C) Pulmonary cytokine concentrations in BALF of challenged mice, as indicated. (D) Number of neutrophils (Gr-1hiCD11bhiF4/80lo) in the BALF wild type C57BL/6, Dectin-1-/- and TLR-4-/- mice sensitized and challenged as in A. Shown are the mean ± SEM of pooled data from three independent experiments (n = 10–12 mice/group). *p<0.05, n.s., not significant. (PDF) [file pone.0134219.s004.pdf]

**A**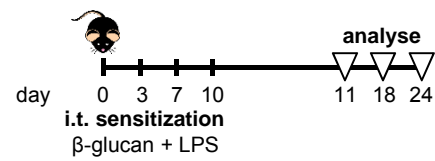**B**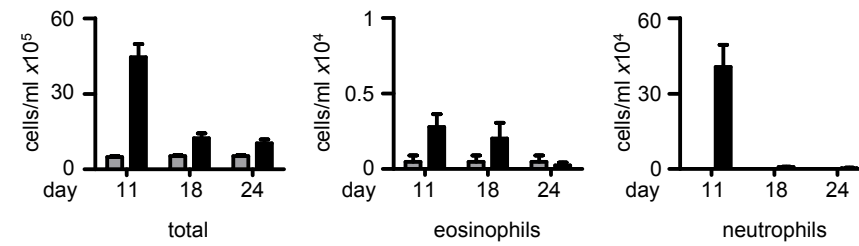

Supplement: S5 Fig — (A) Timeline for sensitization and analysis in C57BL/6 wild type mice with β-glucan (1x10^7^ particles) and LPS (100 ng), as indicated. (B) Airway inflammation in challenged C57BL/6 mice showing the number of total leukocytes, eosinophils (Siglec-FhiGr-1loCD11clo), and neutrophils (Gr-1hiCD11bhiF4/80lo) in the BALF. Shown are the mean ± SEM of pooled data from two independent experiments (n = 4–8 mice/group). (PDF) [file pone.0134219.s005.pdf]

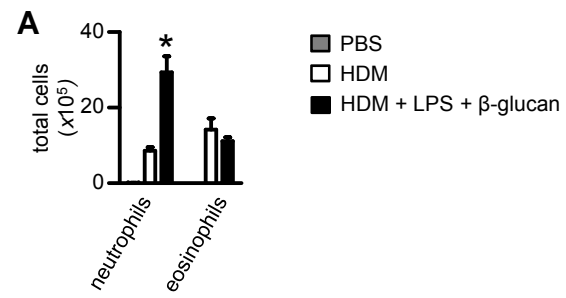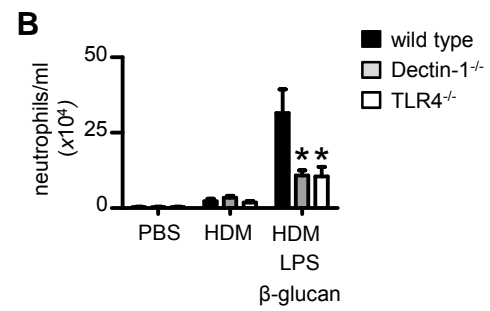

Supplement: S6 Fig — (A) Number of neutrophils (Gr-1hiCD11bhiF4/80lo), inflammatory macrophage/monocytes (F4/80hiCD11bhiGr-1lo) and T cells (CD3hiCD4hi) in whole lungs of mice challenged as in Fig 3. (B) Airway inflammation in challenged wild type C57BL/6, Dectin-1-/- and TLR-4-/- mice sensitized and challenged as in Fig 3, showing the number of neutrophils (Gr-1hiCD11bhiF4/80lo) in the BALF. Shown are the mean ± SEM of pooled data from two independent experiments (n = 8–10 mice/group). (PDF) [file pone.0134219.s006.pdf]

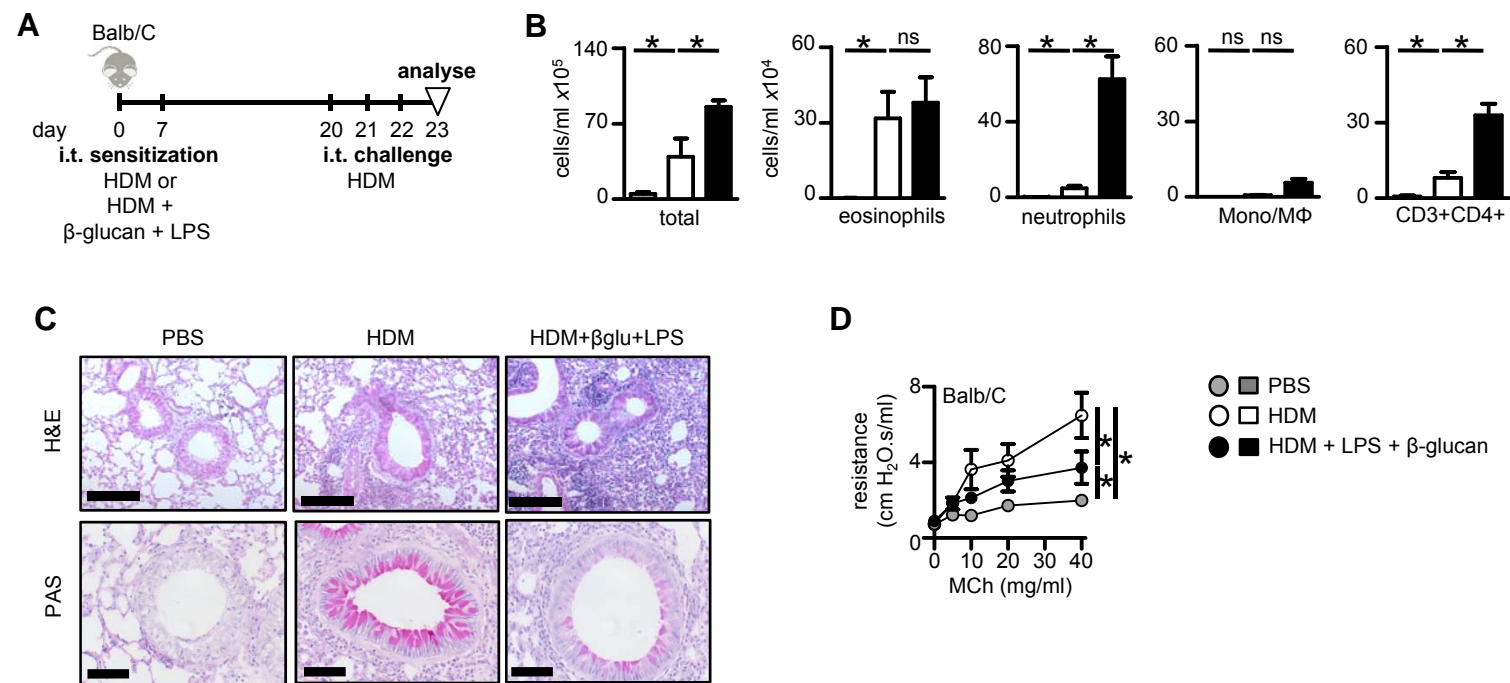

Supplement: S7 Fig — (A) Timeline for HDM sensitization and challenge (10 μg) in Balb/c wild type mice with HDM alone (10 μg) or with the combination of β-glucan (1x10^7^ particles) plus LPS (100 ng), as indicated. (B) Airway inflammation in challenged Balb/c mice showing the number of total leukocytes (left), eosinophils (Siglec-FhiGr-1loCD11clo, middle), and neutrophils (Gr-1hiCD11bhiF4/80lo,right) in the BALF. (C) H&E and PAS stains of formalin fixed lung sections (right) from mice challenged as indicated. Scale bars represent 100 μm (H&E) and 50 μm (PAS). (D) Airway resistance (R) in intubated Balb/c wild type mice exposed to increasing doses of nebulised methacholine (MCh), as indicated. *p<0.05, n.s., not significant. Shown are the mean ± SEM of pooled data from two independent experiments (n = 7–8 mice/group). (PDF) [file pone.0134219.s007.pdf]

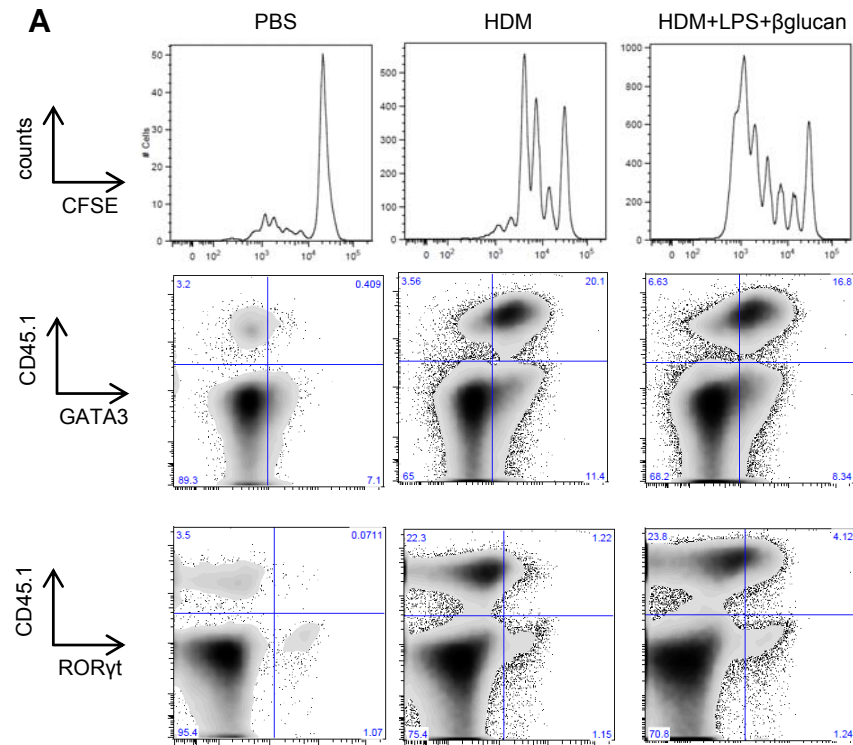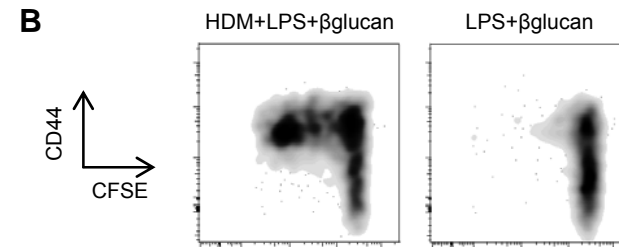

Supplement: S8 Fig — (A) FACS plots representing CD4+ T cell proliferation (CFSE dilution frequency), GATA3 (CD45.1hiGATA3+) and RORγt (CD45.1hiRORγt+) expression in adoptively transferred 1-Derβ T cells (CD4+CD45.1+) isolated from the MLN of mice challenged as in Fig 4. (B) FACS plots showing CD4+ T cell proliferation (CFSE dilution frequency) following sensitization with HDM, β-glucan and LPS or β-glucan and LPS alone, in adoptively transferred 1-Derβ T cells (CD4+CD45.1+) isolated from the MLN of mice challenged as in Fig 4. (PDF) [file pone.0134219.s008.pdf]

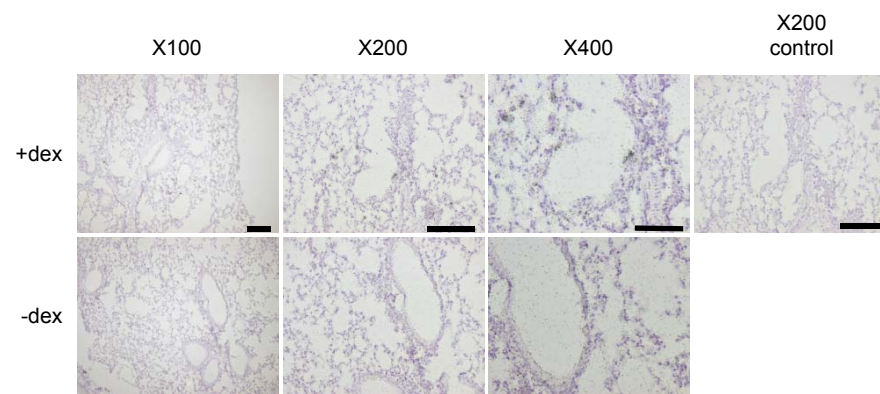

Supplement: S9 Fig — A CCL5-specific riboprobe was hybridized in situ to localize producer cells and signal is evident by collections of black silver grains over individual cells. Shown are micrographs from C57BL/6 wild type mice sensitized with HDM, β-glucan and LPS (100 ng) treated with or without dexamethasone, as indicated, at x100, x200, and x400 magnifications following in situ hybridization with an antisense riboprobe. A control is shown to the right, for which the tissue was probed with a CCL5 sense riboprobe, to show the level of background signal. (PDF) [file pone.0134219.s009.pdf]
